# The 4th Bi-annual international African-Caribbean Cancer Consortium conference: *building capacity to address cancer health disparities in populations of African descent*

**DOI:** 10.1186/1750-9378-9-35

**Published:** 2014-10-22

**Authors:** Elizabeth Blackman, Jasmine Campbell, Carlene Bowen, Ernestine Delmoor, Gilda Jean-Louis, Raphiatou Noumbissi, Yvonne O’Garro, Oni Richards-Waritay, Stanley Straughter, Vera Tolbert, Barbara Wilson, Camille Ragin

**Affiliations:** Cancer Prevention and Control, Fox Chase Cancer Center, Temple University Health System, 333 Cottman Avenuue, Philadelphia, PA 19111 USA; Department of Public Health, Temple University, Philadelphia, PA USA; Thomas Jefferson University Hospital, Philadelphia, PA USA; National Black Leadership Initiative in Cancer - Philadelphia Chapter, Philadelphia, PA USA; Elise Joseph Foundation, Philadelphia, PA USA; Sharing Health and Hope in Cameroon Africa (SHAHICA), Philadelphia, PA USA; St. Vincent and the Grenadines Organization of Pennsylvania, Philadelphia, PA USA; African Family Health Organization (AFAHO), Philadelphia, PA USA; Echoes of Africa, Philadelphia, PA USA; The Coalition of African Communities (AFRICOM), Philadelphia, PA USA; Caribbean Festival & Cultural Organization of Pennsylvania, Philadelphia, PA USA

**Keywords:** Cancer health disparities, Innovative research, Building consortia, Women’s cancers

## Abstract

This is a brief summary of the 4^th^ International Meeting of the African-Caribbean Cancer Consortium (AC3), organized and sponsored by Fox Chase Cancer Center (FCCC), and held on July 21–22, 2012 at the Lincoln University Graduate Center, Lincoln Plaza, Philadelphia, Pennsylvania. AC3 investigators gathered in Philadelphia, PA to present the results of our ongoing collaborative research efforts throughout the African Diaspora. The general theme addressed cancer health disparities and presentations represented all cancer types. However, there was particular emphasis on women’s cancers, related to human papillomavirus (HPV) and human immunodeficiency virus (HIV) infections.

## Introduction

The Fourth International Conference of the African-Caribbean Cancer Consortium (AC3) was held on July 21^st^ and 22^nd^, 2012 at the Lincoln University Graduate Center, Lincoln Plaza at 3020 Market Street, Philadelphia PA, 19104. Conference presentations had a scientific emphasis on women’s cancers with an overarching theme, “*Continuing to Build Capacity to Address Cancer Health Disparities in Populations of African Descent,”* which highlighted the call for high-quality, innovative global cancer research collaborations.

Disparities in access to health care along with environment and host/genetic factors are thought to contribute to the increased cancer risk in minority populations. Among the numerous studies that have investigated gene-environment interactions and cancer risk, most do not include participants of African ancestry; others include small numbers, which results in the inability to report meaningful, generalizable results for this population. This was confirmed by several meta and pooled analyses of the published literature on genetic susceptibility factors and cancer [1–3].

In addition to gene-environment interactions, socioeconomic factors, access to care, lifestyle and other host factors are significant contributors to cancer incidence and mortality. Many studies have sought to address these factors in the African Diaspora, but few studies consider all of these factors in combination.

The current racial disparity in cancer incidence and mortality that exists in the United States has been well described in the literature; the rising burden of cancer is also evident in developing countries [4]. The International Agency for Research on Cancer (IARC) predicted a 40% increase in the number of cancer deaths between 2012 and 2035 in developed countries; for developing countries, more than a 90% increase is expected [5]. Cancer is among the top five leading causes of death in the Caribbean Islands [5]. With a particular focus on women’s cancers, the burden of cervical cancer is highest in Sub-Saharan Africa and the Caribbean [6, 7]. When compared to developed regions of the world, age-adjusted mortality rates are considerably high in both Eastern and Western Africa, where mortality rates were approximately 35 and 25 per 100,000, respectively [6, 8]. Socioeconomic status, advanced disease at diagnosis, and access to effective screening programs are well established risk factors in the increased cancer incidence and mortality among Caribbean and African populations; however, the role in which genetics and environmental exposures play in carcinogenesis within this diverse population is relatively unknown. Therefore, by studying the patterns of cancer incidence and mortality in Caribbean and African populations, we may gain insight on gene-environment interactions related to cancer etiology in these groups.

This meeting focused our attention on the significant need for studies related to cancer in women of African descent, including special considerations for cultural differences and environmental exposures among African-American, African-Caribbean and African populations as a whole. Table 1 summarizes the meeting schedule for both days of the conference. Various topics revolving around cancer research were discussed in hopes to stimulate the sharing of new ideas and the creation of new collaborations. The uniqueness of this conference design was to promote the integration of participants, researchers, students and community partners so as to promote effective knowledge-exchange.

**Table 1:** **Meeting schedule at a glance**

SATURDAY, JULY 21, 2012	
Opening Scientific & Training Conference	
***“Continuing to Build Capacity to Address Cancer Health Disparities in Populations of African Descent”***	
**8:00 – 8:30**	Continental Breakfast
**8:30 – 9:00**	Opening Statements, Welcome
**Keynote Address**	Targeting Women’s Health Disparities: Best-practice Models in Research and Outreach
**9:00 – 9:45**	
**Scientific Session I**	Women’s Cancers in Minority Populations
**9:45 – 10:45**	
**Scientific Session II**	HPV Associated Cancers
**11:00 – 11:45**	
**Cancer Prevention and Research Information**	
***“An ounce of prevention is worth more than a pound of cure!”***	
***(Community Sessions)***	
Lunch N Learn*	Black Women and Cancer: Epidemiology, Research and Progress Made
**11:45 – 1:00**	
Cancer Prevention Workshop I*	Healthy Lifestyles: Reducing Cancer Risk
**1:15 – 1:45**	
Panel Discussion I*	HPV Vaccination Against Cervical Cancer
**1:45 – 2:30**	
**Town Hall Meeting**	Prostate Cancer and Screening, Dispelling the Myths
(Males only)*	
**1:45 – 2:30**	
**Research Skills Workshop I**	Research Methods: Meta and Pooled Analysis
**2:30 – 3:30**	
Cancer Prevention Workshop II*	When Do I Screen?
**2:30 – 3:30**	
Cancer Prevention Workshop III*	How is HIV Related to Cancer?
**3:45 – 4:45**	
Cancer Research Information Session I*	What you Always Wanted to Know About Cancer Research and Clinical Trials
**5:00 – 6:15**	
Cancer Session I*	I Have Cancer, Now What?
**6:15 – 7:15**	
**SUNDAY, JULY 22, 2012**	
***Scientific Training Program (Continued)***	
**7:30 – 8:30**	Continental Breakfast
**Keynote Address**	Cancer Genetics, Genomics and Personalized Medicine
**8:30 – 9:20**	
**Scientific Session III**	Holistic Medicine and Cancer Therapeutics
**9:20 – 10:15**	
**Scientific Session IV**	Cancer in Developing Countries
**10:30 – 11:15**	
**Collaborative Workshop I**	Lunch N Learn & Panel Discussion II: How to Establish Successful Collaborations
**11:15 – 12:15**	
**Scientific Session V**	Building Successful Consortiums
**12:15 – 1:15**	
**Research Skills Workshop II**	Best Practices in Research
**1:15 – 2:15**	
**Scientific Session VI**	NIH Funding Opportunities, National and International
**2:30 – 3:30**	
**Research Skills Workshop III**	Grant Writing: Features of a Successful Grant Application; the Grant Review Process; Mock Study-section Review
**3:30 – 5:00**	
**Scientific Session VII**	Project Development Workshop
**5:15 – 6:15**	
**Closing Remarks and Conference Wrap-Up**	

*Denotes interactive sessions between Community, Scientists & Students.

A groundbreaking component was added to this conference; embedded within the scientific program was a special symposium for community participants. The involvement of community members allowed researchers to better understand the barriers related to minority participation in biomedical research and clinical trials. Furthermore, by attending the conference community members were offered the opportunity to become more knowledgeable about current research progress and the overall importance of research in cancer prevention and treatment. If the community were to become more aware of the positive impact of research on cancer prevention and cancer outcomes, and if this novel interaction were to promote trust of the investigators conducting the research within their community, then persons would be better equipped to recognize the value of participating in biomedical research and/or clinical trials. This was the basis for the integration of community participation into a scientific conference.

To ensure a broad representation of the various African ethnic populations throughout Philadelphia County, individual community leaders and well-established community-based organizations worked in partnership with the AC3 leadership to plan, organize and host the sessions during the community symposium. The organizations included the African Family Health Organization (AFAHO), Caribbean Festival & Cultural Organization of Pennsylvania (“Philadelphia Caribbean Festival Committee”), The Elise Joseph Foundation, The Coalition of African Communities (AFRICOM), National Black Leadership Initiative in Cancer - Philadelphia Chapter, and Sharing Health and Hope in Cameroon Africa (SHAHICA).

The community symposium involved free health screenings and workshops that provided scientific investigators the opportunity to present their research findings to the communities. The workshops were interactive and provided an opportunity for bi-directional knowledge transfer between the community and researchers. These workshops facilitated the setting of research priorities for addressing cancer health disparities. Another vital aspect at the forefront of the 2012 conference was the highlighted importance of cancer registries, especially in the developing countries.

### Saturday July 1^st^ 2012

The opening ceremony was launched by Dr. Robert R. Jennings, (President, Lincoln University, USA) who welcomed all researchers and community leaders to the conference. Dr. Camille Ragin, lead investigator of the AC3, provided a summary of the preceding conferences and the goals moving forward. The Keynote Address was presented by Representative Ronald G. Waters, State Representative for the 191^st^ Legislative District (Delaware and Philadelphia counties, USA). Rep. Waters, who dedicated much of his career to correcting health disparities, spoke on cultural competency and addressed women’s health disparities and the best practice models in research and outreach.

### Scientific presentations

#### Session I: Women’s cancers

The first scientific session was chaired by Dr. Norma McFarlane-Anderson, Professor of Basic Medical Sciences, University of the West Indies, Kingston, Jamaica. Dr. Okey Ibeanu, Gynecologic Oncologist and Assistant Professor at Johns Hopkins University, was the Plenary Speaker for this session. His main research interest is in cervical dysplasia and cancer, as well as outcomes research in minority populations receiving cancer care in the U.S. and abroad. Dr. Ibeanu is actively involved in global health activities in Africa. In Dr. Ibeanu’s address he discussed global and regional epidemiology of cervical cancer. He also described individual and system-related factors that impacted cervical cancer patients in Africa and provided suggestions for improvement. The session concluded with two additional short presentations of selected abstracts by students. The presentations were entitled “Racial/Ethnic Differences in Vitamin D Supplementation among Breast Cancer Survivors” and “Cancer and You: Perspectives from African Women.”

#### Scientific session II: HPV - associated cancers

This session was chaired by Dr. Darron Halliday, Clinical Fellow at the Tom Baker Cancer Center in Calgary, Canada. Dr. Halliday completed his medical training at the University of the West Indies, Mona Kingston, Jamaica. His career goal is to advance women’s cancer care in the Caribbean through collaboration and research with international, government and academic institutions. Dr. Halliday launched the session with a presentation on knowledge and attitudes towards HPV and the HPV vaccines in the Bahamas. His presentation emphasized the need to strengthen preventative programs for HPV infection. He suggested that focused educational programs should be guided by knowledge and attitude-based studies and that reassurance with respect to HPV vaccine safety and efficacy should also be addressed. The session concluded after three short presentations of abstracts: “Knowledge of Cervical Cancer and HPV and Attitudes towards the Use of Vaccines among University Students;” “The Detection of All High Risk Human Papillomavirus DNA in Archival Formalin-Fixed Paraffin-Embedded Cervical Tissue Using Immunohistochemistry and Polymerase Chain Reaction;” and “Comparing Completion and Factors Influencing PAP-testing among African- and Latina-Americans in Two Regions with High Cervical Cancer Incidence.”

#### Research skills workshop I

The Scientific Session for Day 1 concluded with Research Skills Training Workshop I, “Research Methods: Meta and Pooled Analysis,” by AC3 Principal Investigator (PI), Dr. Camille Ragin. The training session provided step-wise instruction on how to conduct a meta-analysis and a pooled analysis. The presentation also highlighted the advantages and disadvantages of each type of analysis and summarized the software used to generate data.

### Community symposium

Two types of workshops were offered during the second half of the day; cancer prevention, and cancer research information. Community participants were informed of various research methods, the appropriate time to screen, how HIV is related to cancer, and clinical trials.

The Community Symposium began at noon with the Lunch N Learn keynote presentation by Dr. Raleigh Butler, Director of Gynecologic Oncology, Princess Margaret Hospital, Nassau, Bahamas. Dr. Butler’s presentation, “Black Women and Cancer: Epidemiology, Research and Progress Made,” highlighted healthcare disparities related to common gynecologic cancers and shared facts about endometrial, ovarian, lung and breast malignancies. His presentation highlighted the progress made by the AC3 and other national, regional and global entities and also discussed future directions for AC3.

#### Cancer prevention workshop I

The first community workshop, entitled “Healthy Lifestyles: Reducing Cancer Risk,” was hosted by The Coalition of African Communities-Philadelphia. The featured speaker, Carlene Bowen, Registered Transplant Dietician at Thomas Jefferson University Hospital, Philadelphia, PA, helped the audience identify modifiable and non-modifiable risk factors associated with cancer development. Ms. Bowen discussed the link between diet, physical activity and health-promoting behaviors, with certain types of cancers.

#### Cancer prevention workshop II

The second community workshop, entitled “When do I Screen?” was hosted by the African Family Health Organization (AFAHO). Dr. Deborah K. Witt, Assistant Professor in the Department of Family and Community Medicine at Thomas Jefferson University Hospital, Philadelphia, PA, presented on the importance of screening for the African American community. Dr. Witt’s presentation defined proper screening habits and reviewed established recommendations for screening for colon, cervical, prostate and breast cancer.

#### Cancer prevention workshop III

Sharing Health and Hope in Cameroon Africa (SHAHICA) hosted the final cancer prevention workshop of the community symposium. Dr. Ngozi Onuoha, a geriatric internist within the University of Pennsylvania Health System, Philadelphia, PA, presented on behalf of Dr. Helen Kwa kwa, Director of HIV Clinical Services at the Philadelphia Department of Health. The goal of the presentation, entitled “How is HIV Related to Cancer?” was to highlight the risk of cancer within the HIV-infected population. Some questions that were addressed throughout the presentation were: 1) Why do people living with HIV have a higher risk of cancer? 2) Has the introduction of antiretroviral treatment changed the risk of developing cancer for people infected with HIV? and 3) What can people living with HIV do to reduce their risk of cancer or to detect cancer early?

#### Cancer research information session I

The Philadelphia Caribbean Festival Committee hosted the symposium’s cancer research session. The session included two presentations: “Principles of Cancer Research,” presented by AC3 PI Camille Ragin, and “What You Always Wanted to Know about Cancer Research and Clinical Trials, But Did Not Know Who to Ask!” presented by Ernestine P. Delmoor, MPH, President of the National Black Leadership Initiative in Cancer-Philadelphia Chapter, PA. Dr. Ragin’s presentation gave an overview on the different types of study designs used in cancer research, emphasizing the impact of cancer research on improving health and the importance of minority participants in research studies. Ms. Delmoor began an open discussion that focused around medical research in the minority community and whether or not there are collective health benefits for minorities to participate in clinical trials. Ms. Delmoor’s presentation aimed to help community members become more comfortable talking about cancer research and clinical trials. The presentation enabled the audience to make the connection between health care, clinical trials and racial disparities.

### Satellite meeting I

While this AC3 conference focused on women’s cancers, the meeting organizers noted the significant impact that prostate cancer has on women and their families. Therefore, a special session was organized to address this important men’s health topic. A Town Hall meeting for men was held as a satellite session concurrently scheduled with Cancer Prevention Workshop III and Cancer Research Information Session I. The meeting was designed for male participants only to discuss prostate cancer, screening and dispel myths. The Town Hall meeting was facilitated by prostate cancer survivor and radio personality of Philadelphia’s 105.3 WDAS, Mr. Doug Henderson. The panelists included behavioral scientist Dr. JoAnn Oliver (USA), urologists Drs. Robin Roberts (Bahamas) and Robert Yearwood (Grenada), community advocate and prostate cancer survivor Robert Nelson (USA), as well as radiation oncologist, Dr. Curtland Deville (USA).

### Sunday July 22^nd^ 2012

Dr. Carolyn Fang, Co-Leader, Cancer Prevention and Control, Fox Chase Cancer Center, USA, and Dr. Camille Ragin, launched Day 2 of the conference with opening remarks. Dr. Jose Russo, Director, Breast Cancer Research Laboratory and the NCI-NIEHS Breast Cancer and the Environment Research Center, Fox Chase Cancer Center, followed with the Keynote Address. Dr. Russo’s presentation addressed a genomic personalized approach to breast cancer prevention. This was followed by a featured abstract presentation by Dr. Renee Reams, Florida A&M University. Dr. Reams’s talk provided an example of identifying key pathways important in aggressive cancer using a genomics approach.

#### Scientific session III: holistic medicine and cancer therapeutics

The first general scientific session, “Holistic Medicine and Cancer Therapeutics,“ was chaired by Dr. Maria Jackson, Senior Lecturer in the Department of Community Health and Psychiatry at the University of the West Indies, Mona in Kingston, Jamaica. The session consisted of two presentations by Drs. Lawrence Williams from the University of the West Indies, Mona, Jamaica, and Esther Matu from the Kenya Medical Research Institute. Dr. Williams presented information on Dibenzyl trisulfide (DTS), a cytotoxic agent isolated from *Petivera alliacea* and its effects on neuroblastoma, breast and ovarian cancers. DTS is derived from the Guinea hen weed, has also been used for enhancing the immune system, treating diabetes, improving memory, and as an anti-inflammatory. Dr. Williams explained that DTS exhibits potent anti-proliferation/cytotoxic activity on a wide range of cancer cell lines specifically through a cytokine switching mechanism that down-regulates cytokines in the T1 helper cells (cytokines that have pro-inflammatory properties) and up-regulates those in the T2 helper cells. Dr. Matu presented information on African traditional medicine (ATM) in the management of cancers. Dr. Matu’s presentation began with a detailed description of ATM and how it should be considered an indigenous system of healthcare and not complimentary and/or alternative health care. Dr. Matu provided the audience with several examples of plants and herbs that are currently being used in traditional medicine practices in Kenya, which included Prunus *Africana,* used in the treatment of benign prostate hyperplasia; and *Vernonia spp,* used in the treatment of oesophageal cancer. Dr. Matu stressed that most remedies used in ATM have not reached the clinical evaluation stage, but that it was important to note that yesterday’s folk remedies may one day be what the 21^st^ century doctor orders.

#### Scientific session IV: cancer in developing countries

The second general scientific session for Day 2 focused on cancer in developing countries and was moderated by Dr. Samuel Gathere, Consultant Otolaryngologist and Research Officer at the Kenya Medical Research Institute. Dr Gathere started the session by describing cancer statistics from the National Cancer Registry in Kenya where breast and cervical cancers were most prominent among women. The session continued with presentations from three Caribbean Cancer Registries as well as a presentation by Dr. Belinda Morrison, who highlighted the impact of the National Health Fund policy on cancer treatment in Jamaica. Although Dr. Morrison’s presentation focused on prostate cancer rather than women’s cancer, it provided a good example of the impact of health policy decisions in developing countries on facilitating greater access to newer types of treatment, thus ultimately improving health outcomes.

#### Afternoon sessions

The second half of Day 2 focused on sessions geared toward research capacity building that will help to sustain global cancer research. A panel discussion was facilitated by Dr. Robin Roberts on “How to Establish Successful Collaborations.” The panelists included community leaders Dr. Vera Tolbert from Philadelphia and Dr. Delroy Louden from the Caribbean, as well as researchers Dr. Renee Reams from the U.S. and Dr. Dionne Dames from the Caribbean. A session on “Building Successful Consortia” provided presentations from leaders of three global consortia − Dr. Folakemi Odedina, University of Florida, representing the Prostate Cancer Transatlantic Consortium (CaPTC), Dr. Timothy Rebbeck, University of Pennsylvania, representing the Men of African Descent and Carcinoma of the Prostate (MADCaP), and Dr. Camille Ragin, Fox Chase Cancer Center, representing the African-Caribbean Cancer Consortium (AC3).

#### Research skills workshop II

This workshop was facilitated by Theresa Berger, MBE, Director of Academic Affairs, Fox Chase Cancer Center, Philadelphia, PA provided instructions on best practices in research. The presentations highlighted hypothesis development, research study design, planning and management of a research study, ethics and responsible conduct of research. Additional areas covered in this workshop were health literacy and communications, planning and organizational skills in community outreach, cultural competency and community-based interventions.

Research Skills Workshop III was presented by Dr. Folakemi Odedina, University of Florida, who provided instruction on grant writing and features of a successful grant application. Scientific Session VI provided information on funding opportunities: local, national and international. The presentations were made by Dr. Nina Ahmad from the Philadelphia Foundation and Dr. Damali Martin from the National Cancer Institute, Epidemiology and Genomics Research Program, as well as Dr. John Flanigan from the National Cancer Institute, Center for Global Health. Session VII, the Project Development Workshop, was chaired by Ms. Theresa Berger, Fox Chase Cancer Center, during which pilot projects were presented by junior investigators in order to gain feedback from senior research investigators.

### Satellite meeting 2

A meeting hosted by the National Cancer Institute, Center for Global Health was held concurrently with the Project Development Workshop and involved all of the cancer registrars that attended the conference. The meeting was to discuss the National Cancer Institute, Center for Global Health’s vision for building capacity for cancer registration in the Caribbean. Countries represented included Anguilla, Grenada, Guyana, Jamaica and Martinique. The need to establish a regional cancer registry in the Caribbean was agreed upon by all in attendance, and a future meeting that would include stakeholders, cancer registrars and representatives of the Ministries of Health was recommended.

Throughout the conference, participants (community, researchers and students) discussed the status of the ongoing collaborative studies and defined research priorities for future studies. The conference concluded with closing remarks by Dr. Camille Ragin.

### Conference evaluations

Evaluation forms were distributed for both conference days. For each session, the relevance of the topic, quality and speaker presentations, and knowledge gained were evaluated. Attendees had the opportunity to answer questions rating 1–5 (1 = lowest rating and 5 = highest rating) Participants were also asked to provide their comments and feedback in short answers. Attitudes toward venue and session length were also rated 1–5.

**Figure 1:**
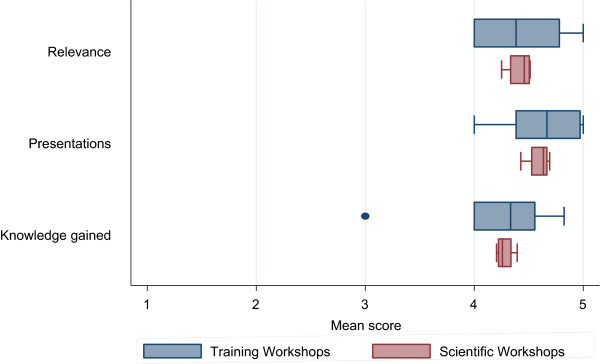
**Box plots showing participant mean scores of each scientific (blue) and training (pink) session according to relevance, presentations and knowledge gained (ranked 1 low - 5 high).** Error bars refer to the range of mean scores, and the vertical line within each box plot refers to the mean of the mean scores.

All sessions were grouped into broad categories (Scientific, Training or Community). Mean scores (participant score ÷ total number of participants) were calculated for each session. An overall mean score for each broad category was determined by calculating the mean of means for each session within each category (session score ÷ total number of sessions). Figure 1 summarizes the mean evaluation scores for the scientific and training workshops based on relevance, presentations and knowledge gained. In the scientific workshop, the number of participants that completed the evaluation surveys in each session varied from 36 to 43 persons. In the training workshop, the number of completed evaluation surveys obtained varied from one to 34 in each session. On average, participants felt that the topics presented during the sessions were quite relevant and the presentations were above average. Based on their knowledge prior to attending each session, participants indicated that they learned a lot.

**Figure 2:**
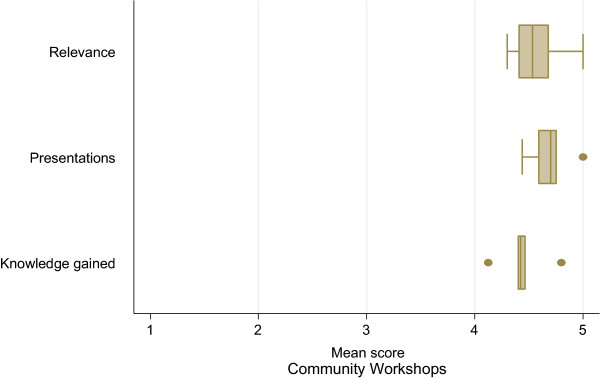
**Box plots showing participant mean scores for each community session according to relevance, presentations and knowledge gained (ranked 1 low - 5 high).** Error bars refer to the range of mean scores, and the vertical line within each box plot refers to the mean of the mean scores.

Figure 2 summarizes the mean evaluation scores for the community workshops based on relevance, presentations and knowledge gained. The number of completed evaluation surveys obtained varied from 6 to 28 in each session. Similar to the scientific workshops, community participants felt that the topics presented were quite relevant and the presentations were above average. Based on their knowledge prior to attending each session, participants indicated that they learned a lot.

There were approximately 20 male participants at the Town Hall meeting. The men felt that the session was very effective (overall mean rating = 4.6, mean rating relevance = 4.7, mean rating presentations/discussion = 4.5 and mean rating knowledge gained = 4.7). The participants were asked to comment on the most beneficial aspects of the Town Hall meeting. Comments indicated that discussion surrounding prostate cancer statistics and the realization that research has succeeded in convincing Black men to screen for prostate cancer were most beneficial.

Based on the participants’ comments overall, enthusiasm was overwhelming. There were references to the superior quality of the keynote speakers and presentations. Participants were also asked to comment on ways the conference could be improved. There was an overwhelming request for more time given for question and answers at the end of each session. Educational brochures could have been more helpful for some sessions, and one participant indicated that there were too many sessions in a single day. One participant indicated that a future workshop on “how to build a cancer registry” would have been very informative.

There were a number of take home messages from the various sessions. 1) For additional impact there appeared to be an urgent need for closer collaboration between the existing cancer registries in the Caribbean region, 2) there is a need for continued community outreach and cancer education in the African Diaspora, 3) the type of challenges related to sustaining cancer screening programs involving HPV testing and/or Pap testing vary across different low-resource countries, and 4) cultural differences impact cancer risk in the African Diaspora.

To our knowledge, this conference was the first of its kind to integrate community-oriented sessions focused on the African and Caribbean Diaspora within a traditional scientific conference. The community aspect of the conference was a success. Community members were able to gain valuable information on the importance of their roles in cancer prevention and cancer treatment research. The community sessions also allowed community members to convey, to the scientists, their fears, concerns, and other hindrances that limit their willingness to participate in research.
